# The PCI domains are “winged” HEAT domains

**DOI:** 10.1371/journal.pone.0268664

**Published:** 2022-09-12

**Authors:** Eleanor Elise Paul, Assen Marintchev

**Affiliations:** Department of Physiology & Biophysics, Boston University School of Medicine, Boston, Massachusetts, United States of America; Karl-Franzens-Universitat Graz, AUSTRIA

## Abstract

The HEAT domains are a family of helical hairpin repeat domains, composed of four or more hairpins. HEAT is derived from the names of four family members: **h**untingtin, eukaryotic translation **e**longation factor 3 (eEF3), protein phosphatase 2 regulatory **A** subunit (PP2A), and mechanistic **t**arget of rapamycin (mTOR). HEAT domain-containing proteins play roles in a wide range of cellular processes, such as protein synthesis, nuclear transport and metabolism, and cell signaling. The PCI domains are a related group of helical hairpin domains, with a “winged-helix” (WH) subdomain at their C-terminus, which is responsible for multi-subunit complex formation with other PCI domains. The name is derived from the complexes, where these domains are found: the 26S **P**roteasome “lid” regulatory subcomplex, the **C**OP9 signalosome (CSN), and eukaryotic translation **i**nitiation factor 3 (eIF3). We noted that in structure similarity searches using HEAT domains, sometimes PCI domains appeared in the search results ahead of other HEAT domains, which indicated that the PCI domains could be members of the HEAT domain family, and not a related but separate group, as currently thought. Here, we report extensive structure similarity analysis of HEAT and PCI domains, both within and between the two groups of proteins. We present evidence that the PCI domains as a group have greater structural similarity with individual groups of HEAT domains than some of the HEAT domain groups have among each other. Therefore, our results indicate that the PCI domains have evolved from a HEAT domain that acquired a WH subdomain. The WH subdomain in turn mediated self-association into a multi-subunit complex, which eventually evolved into the common ancestor of the Proteasome lid/CSN/eIF3.

## Introduction

Helical repeat domains are widespread in eukaryotic genomes, and are involved in virtually every major cellular process. These encompass several families, including tetratricopeptide repeat (TPR), ankyrin repeat (ANK), armadillo (ARM), as well as the HEAT repeat domain family (reviewed in [1–4]). The acronym HEAT is derived from the names of several founding members of the family: **h**untingtin, eukaryotic translation **e**longation factor 3 (e**E**F3), protein phosphatase 2 regulatory **A** subunit (PP2**A**), and mechanistic **t**arget of rapamycin (m**T**OR) [5]. Of these families, ARM and HEAT repeat domains are only found in eukaryotes, and likely arose more recently during evolution [2]. Another, smaller group of eukaryote-specific helical repeat proteins are the PCI domains. Most PCI domain-containing proteins are subunits of the **P**roteasome “lid” regulatory subcomplex, the **C**OP9 signalosome (**C**SN), or eukaryotic translation **i**nitiation factor 3 (e**I**F3) [6–9]. The Evolutionary Classification of Protein Domains (ECOD) database (http://prodata.swmed.edu/ecod/) [10, 11] groups the HEAT and PCI domains in the ARM-repeat H-group, along with multiple other helical repeat domain proteins.

Unlike most globular domains, helical repeat domains do not have a fixed size, with a distinct beginning and end. Instead, they consist of varying numbers of helical repeats. The larger domains have varying curvatures and tend to form solenoids. This plasticity likely contributed to the fast divergence of helical repeat domains in size, structure, sequence, and function. Sequence analyses have revealed that it is often impossible to detect statistically significant sequence similarity, even among members of the same domain family. The variable length, irregular shapes, and low/undetectable sequence similarity have made the analysis of the evolution of these domain families challenging. Sequence conservation is limited to the interfaces between adjacent helices and tends to be lower than for other domain families (reviewed in [1, 3, 5]).

**Fig 1:**
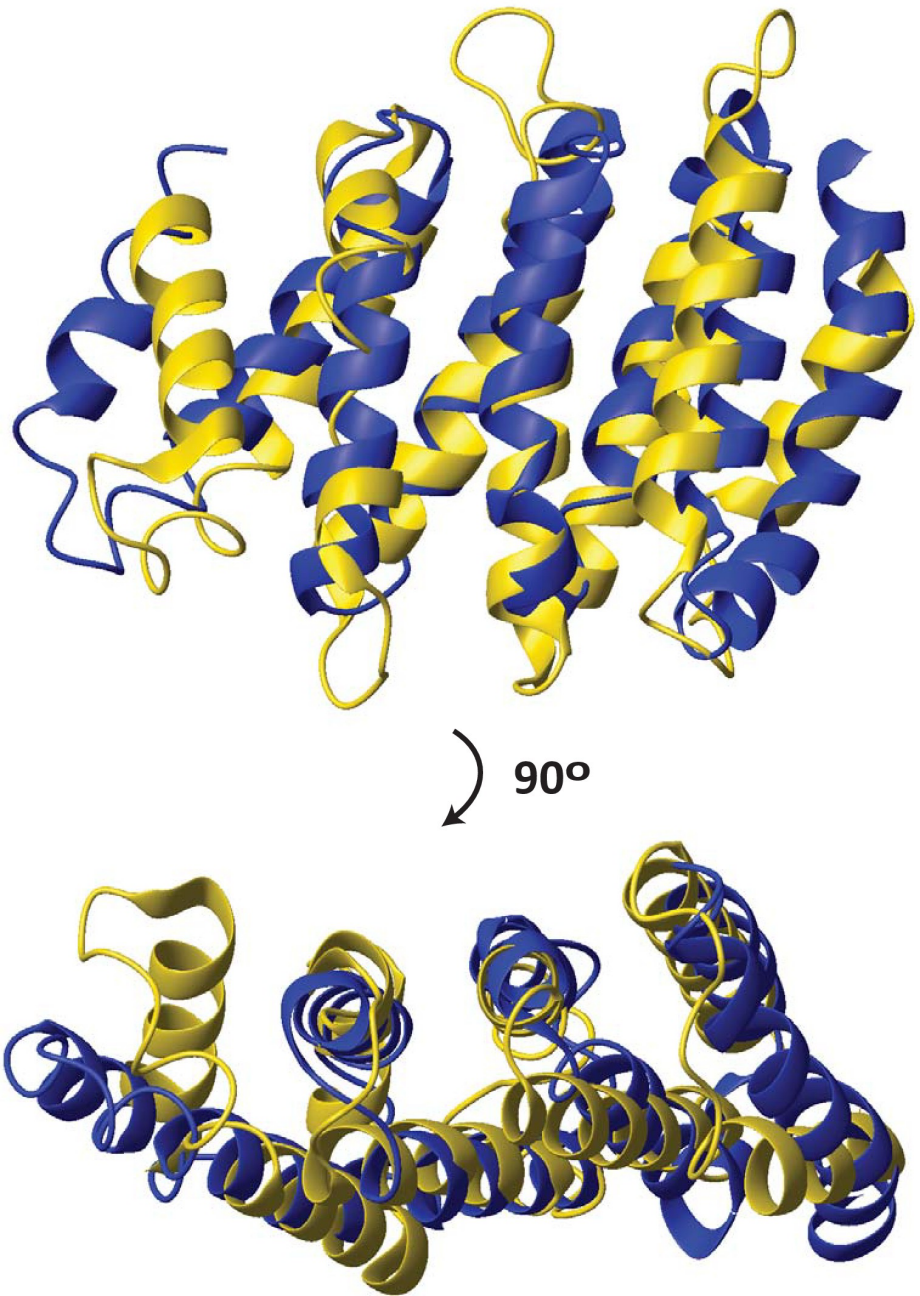
Structure alignment between the HEAT domain of CTF3 and the PCI domain of RPN12. DALI [27]-based structure alignment between the HEAT domain of *S*. *cerevisiae* CTF3 (6wuc.pdb, chain B) and the PCI domain of human RPN12 (5l4k.pdb, chain P). The structures are shown in ribbon. CTF3 is colored gold; RPN12 is colored blue. Only the aligned portions of the two structures are shown.

The HEAT repeat domain family was first reported in 1995 [5]; these domains consist of a series of helical hairpins, each formed by two antiparallel helices, packing against each other and the two surrounding helical hairpins (Fig 1). The number of hairpins varies from as few as four to over 50 [5, 12–14].

Along with another domain, MPN/Mov34, the PCI domain was discovered as a structural unit found in three multi-subunit complexes, 26S Proteosome lid, CSN, and eIF3 [15–18]. PCI domains have very similar organization to the HEAT domains and the other helical repeat domains, but also have a “winged-helix” (WH) subdomain at their C-terminus. The WH subdomains mediate complex assembly with the WH subdomains of other PCI domain containing subunits. The 26S Proteosome lid, CSN, and eIF3 are multiprotein complexes with a common ancestor and similar organization, including the presence in each of them of six PCI domain-containing subunits: RPN3, RPN5, RPN6, RPN7, RPN9, and RPN12 in the Proteasome lid; CSN3, CSN4, CSN2, CSN1, CSN7, and CSN8 in the CSN; and eIF3l, eIF3a, eIF3c, eIF3e, eIF3m, and eIF3k in eIF3, respectively, as well as two MPN/Mov34 domain-containing subunits. While the MPN domains show high degree of sequence conservation, this is not the case for the PCI domains. For PCI domain-containing subunits in the Proteasome lid and the CSN complexes, the pairwise correspondence was possible to establish based on sequence similarity. However, similarity was only detected to two of their eIF3 counterparts (among CSN3, eIF3l, and RPN3, and among CSN3, eIF3k, and RPN12). For the others, the pairwise correspondence only became obvious when the structures of the Proteasome, CSN, and eIF3 became available. In each of these complexes, the WH subdomains form a ring in the core of the complex. No similarity was detected between the six groups of PCI domain proteins [6–8, 16–18]. Exhaustive PSI-BLAST [19] searches yielded additional pairs of PCI domains with significant sequence similarity; while using HHblits [20], we were able to detect statistically significant sequence similarity among all PCI domains, but not between PCI and HEAT domains, or between different groups of HEAT domains (data not shown). eIF3 in some groups of organisms has lost part of its subunits and has fewer than six PCI domains. For example, *Saccharomyces cerevisiae (S*. *cerevisiae)* has two of the PCI subunits: eIF3a and eIF3c; while *Schizosaccharomyces pombe (S*. *pombe)* has four: eIF3a, eIF3c, eIF3e, and eIF3m subunits [15, 21–26].

The structure similarity between HEAT and PCI domains is illustrated in Fig 1 and has been noted previously [9], although no statistically significant sequence similarity could be detected. However, as pointed out above, there is no detectable sequence similarity among distant members of the HEAT family, either [8, 17]. It is of course, theoretically possible that the PCI domains and the different groups of HEAT domains with no detectable sequence similarity could be the product of convergent evolution because of the simple repetitive nature of the helical hairpins.

When performing DALI [27] searches with HEAT domains, we noticed that sometimes PCI domain structures appeared in the search results with higher scores that some of the known HEAT domains. This prompted us to investigate whether the PCI domains are a subset of the HEAT family, or the PCI and HEAT domains are two related but distinct families. Here we performed extensive comparative structure similarity analysis, which demonstrates that the PCI domains are a sub-family of the HEAT domains, which originated when a HEAT domain acquired a WH domain at its C-terminus.

## Results

**Fig 2:**
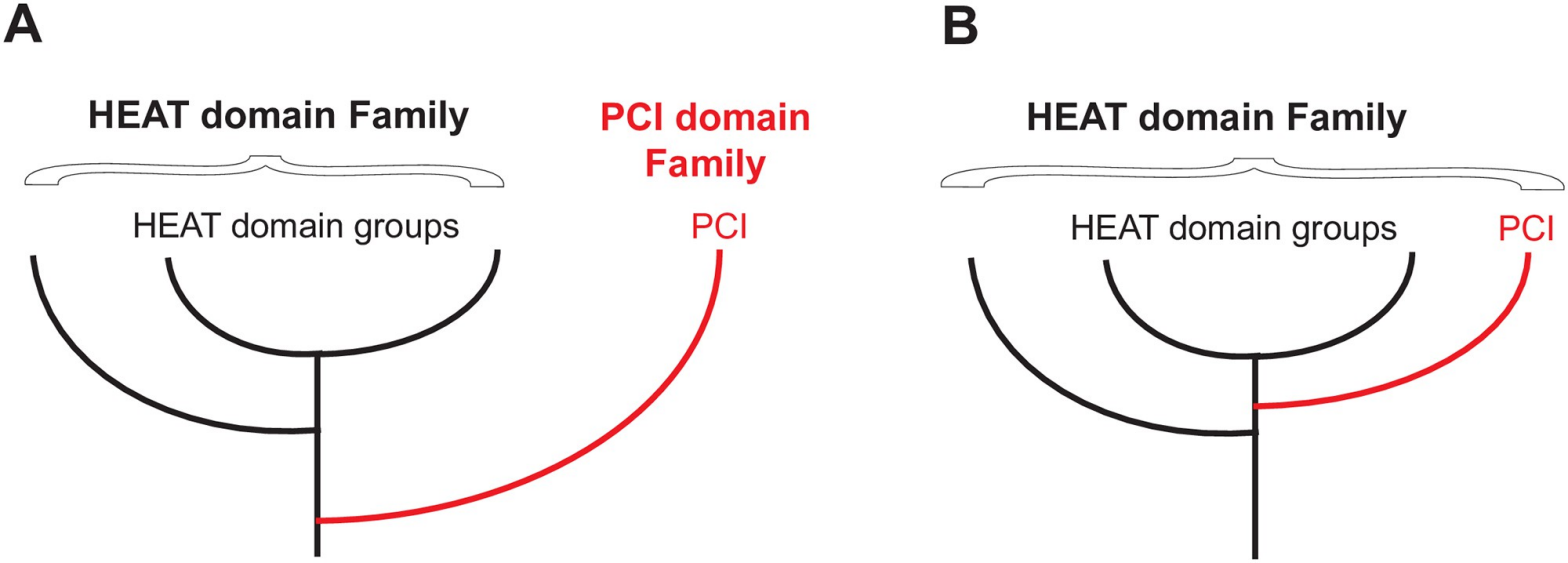
Possible alternatives for the evolution of PCI domains. **A**. PCI domains (red) diverged from HEAT domains before the last common ancestor of all HEAT domains and constitute a separate family of domains, as currently thought. **B**. The PCI domains are members of the HEAT domain family that acquired a WH subdomain after the last common HEAT domain ancestor.

We reasoned that if structure similarity between the PCI domains and individual groups of known HEAT domains is comparable to the structure similarity among the groups of HEAT domains, then the PCI domains are a group of HEAT domains that acquired a WH subdomain during evolution, after the HEAT domains had diverged from other helical repeat domain families. Conversely, if all groups of HEAT domains have greater structure similarity to each other than to the PCI domains, then the PCI domains split earlier, before the common ancestor of all HEAT domains (Fig 2):

- If the PCI domains have lower structure similarity scores with all groups of HEAT domains than the scores among HEAT domain groups, then the PCI domains split off from their common ancestor with HEAT domains before the individual HEAT domain groups diverged from each other (Fig 2A).
- If the average similarity scores between PCI domains and at least one group of HEAT domains are comparable, or higher, than the lowest average similarity scores between at least two groups of HEAT domains, then the PCI domains are a group of HEAT domains that have acquired a winged helix (Fig 2B).

To account for the presence of a Winged Helix (WH) subdomain in PCI domains, but not in the HEAT domains, which could affect the structure similarity scores, we also used PCI domain structures with deleted WH subdomains. Finally, helical repeat domains pose unique challenges for structure similarity software, because they have a repetitive structure, a variable number of repeats, and varying curvature. To try to compensate partially for these factors, we also used four-hairpin fragments of HEAT and PCI domains.

We performed extensive structure similarity searches with HEAT and PCI domains with known structures, using the DALI server [27]. The searches with a HEAT domain structure consistently yielded PCI domains with structure similarity scores (Z-scores) comparable to those of other HEAT domains, and *vice versa*. The PCI domain structures were the only group that consistently appeared in the searches with high scores. All further analysis was focused on HEAT and PCI domain structures only; however, there could be other groups of domains with comparable structure similarity to HEAT domains. We selected a representative set of structures with high-resolution and avoiding close relatives, and then subdivided the representative structures into groups based on structure similarity scores. The HEAT domains fell into four groups, based on structure similarity, as well as a couple of outlier structures, which were considered as a fifth group. (**Table in** S1 Table) The “founding” members of the HEAT domain family, including Huntingtin, eEF3, and PP2A [5], were in Group 1. The MA3, MIF4G, and W2 domains [12–14, 28–31] formed individual groups, Groups 2, 3, and 4, respectively. Finally, two of the selected proteins, UTP10 and the FAT (**F**RAP, **A**TM, **T**RRAP) HEAT repeat domain of mTOR [32, 33], distinct from the “classical” HEAT repeat domain in the N-terminal portion of the protein [5], were outliers and placed in Group 5. The PCI domains formed Group 6.

**Fig 3:**
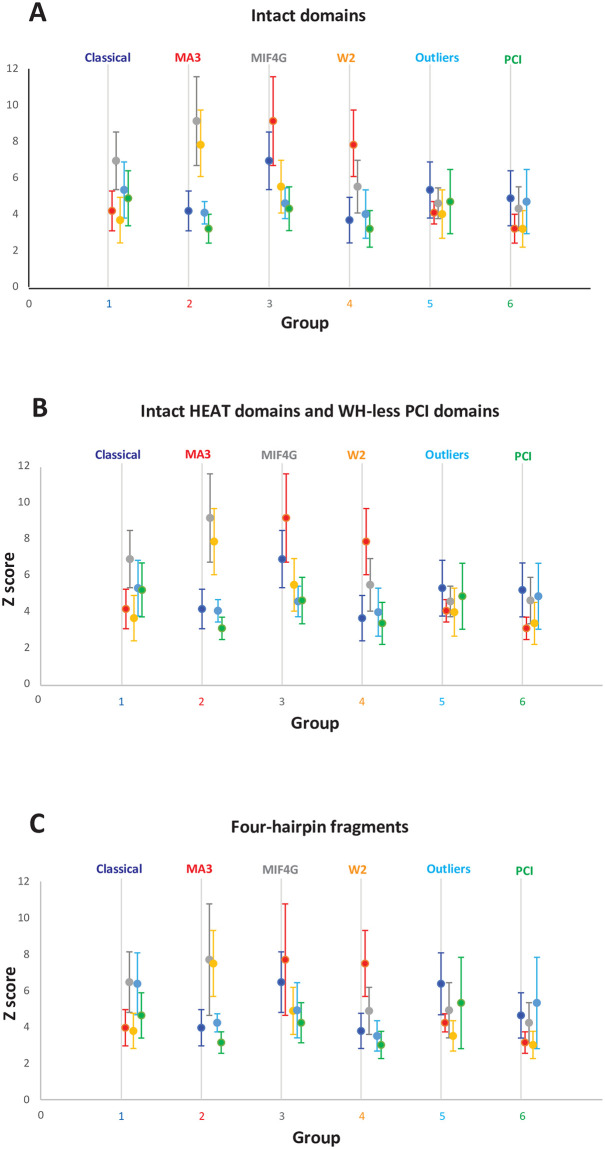
Structure similarity scores among groups of HEAT and PCI domains. Average pairwise DALI [27] Z-scores +/- standard deviation (SD) between members of individual HEAT and PCI domain groups. Groups are labeled and color-coded. The Z-scores of each group with the other five groups are shown on the Y-axis, with slight offset along the X-axis, and color-coded. Classic HEAT domains (Group 1) are navy. MA3 domains (Group 2) are red. MIF4G domains (Group 3) are grey. W2 domains (Group 4) are orange. Outlier HEAT domains (Group 5) are light blue. PCI domains are green. Intragroup Z-scores are not shown.

We found that the average similarity score between Group 1 HEAT domains and PCI domains (Group 6), was 4.8 (where scores above 2 indicate similarity). This score was higher than those between Group 1 and Groups 2 (4.2) and 4 (3.6). Similar results were observed for the outlier HEAT domains (Group 5): average similarity score with the PCI domains (Group 6) was 4.6, whereas those with Groups 2 and 4 were both 3.9. The similarity score of Group 5 with Group 3 was 4.5, comparable to that with Group 6 (PCI) (Table 1, Fig 3A). This observation indicates that the PCI domains belong to the family of HEAT domains.

**Table 1:** Structure similarity scores among groups of HEAT and PCI domains.

	Group 1	Group 2	Group 3	Group 4	Group 5	Group 6
	Classical HEAT	MA3	MIF4G	W2	HEAT Outliers	PCI
**A. Intact structures**						
**Vs. group 1**	11.1 +/- 2.8	4.1 +/- 1.1	6.8 +/- 1.6	3.6 +/- 1.2	5.2 +/- 1.5	4.8 +/- 1.5
**Vs. group 2**	4.1 +/- 1.1	12.9 +/- 4.1	9.0 +/- 2.4	7.8 +/- 1.8	4.0 +/- 0.6	3.1 +/- 0.8
**Vs. group 3**	6.8 +/- 1.6	9.0 +/- 2.4	13.6 +/- 3.7	5.4 +/- 1.4	4.5 +/- 0.8	4.2 +/- 1.2
**Vs. group 4**	3.6 +/- 1.2	7.8 +/- 1.8	5.4 +/- 1.4	12.7 +/- 1.6	3.9 +/- 1.3	3.1 +/- 1.0
**Vs. group 5**	5.2 +/- 1.5	4.0 +/- 0.6	4.5 +/- 0.8	3.9 +/- 1.3	5.7 +/- 0.0	4.6 +/- 1.7
**Vs. group 6 (PCI)**	4.8 +/- 1.5	3.1 +/- 0.8	4.2 +/- 1.2	3.1 +/- 1.0	4.6 +/- 1.7	10.6 +/- 3.8
**B. Wingless PCI**						
**Vs. group 1**	11.1 +/- 2.8	4.1 +/- 1.1	6.8 +/- 1.6	3.6 +/- 1.2	5.2 +/- 1.5	5.1 +/- 1.5
**Vs. group 2**	4.1 +/- 1.1	12.9 +/- 4.1	9.0 +/- 2.4	7.8 +/- 1.8	4.0 +/- 0.6	3.1 +/- 0.6
**Vs. group 3**	6.8 +/- 1.6	9.0 +/- 2.4	13.6 +/- 3.7	5.4 +/- 1.4	4.5 +/- 0.8	4.6 +/- 1.3
**Vs. group 4**	3.6 +/- 1.2	7.8 +/- 1.8	5.4 +/- 1.4	12.7 +/- 1.6	3.9 +/- 1.3	3.3 +/- 1.1
**Vs. group 5**	5.2 +/- 1.5	4.0 +/- 0.6	4.5 +/- 0.8	3.9 +/- 1.3	5.7 +/- 0.0	4.8 +/- 1.8
**Vs. group 6 (PCI)**	5.1 +/- 1.5	3.1 +/- 0.6	4.6 +/- 1.3	3.3 +/- 1.1	4.8 +/- 1.8	7.9 +/- 2.8
**C. Four-hairpin fragments**						
**Vs. group 1**	9.1 +/- 2.4	3.9 +/- 1.0	6.4 +/- 1.6	3.7 +/- 1.0	6.3 +/- 1.7	4.6 +/- 1.2
**Vs. group 2**	3.9 +/- 1.0	10.8 +/- 3.2	7.6 +/- 3.0	7.4 +/- 1.8	4.2 +/- 0.5	3.1 +/- 0.6
**Vs. group 3**	6.4 +/- 1.6	7.6 +/- 3.0	10.3 +/- 4.5	4.8 +/- 1.3	4.8 +/- 1.5	4.2 +/- 1.1
**Vs. group 4**	3.7 +/- 1.0	7.4 +/- 1.8	4.8 +/- 1.3	15.5 +/- 3.6	3.5 +/- 0.8	3.0 +/- 0.7
**Vs. group 5**	6.3 +/- 1.7	4.2 +/- 0.5	4.8 +/- 1.5	3.5 +/- 0.8	5.6 +/- 0.0	5.2 +/- 2.5
**Vs. group 6 (PCI)**	4.6 +/- 1.2	3.1 +/- 0.6	4.2 +/- 1.1	3.0 +/- 0.7	5.2 +/- 2.5	7.6 +/- 2.3

Average pairwise DALI [27] Z-scores +/- standard deviation (SD) between members of individual HEAT and PCI domain groups. The average intragroup Z-scores (in the diagonal) are highlighted in grey.

The same conclusion was reached when using a set of PCI domains with deleted WH subdomain, which tended to increase further the similarity scores between the PCI domains (Group 6) and the HEAT domain groups. The score between Group 1 and Group 6 was now 5.1, and that between Group 5 and Group 6 was now 4.8. Furthermore, the new score between Group 3 and Group 6 was now 4.6, comparable to that between Group 3 and Group 5 (4.5) (Table 1, Fig 3B).

Finally, we used a set of protein fragments with only four helical hairpins, so that all structures had the same size. Again, the average similarity score between Group 1 HEAT domains and PCI domains (Group 6), 4.6, was higher than those between Group 1 and Groups 2 (3.9) and 4 (3.7). Likewise, the average similarity score of Group 5 with the PCI domains (Group 6), 5.2, was higher than those with Group 2 (4.2), Group 3 (4.8), and Group 4 (3.5) (Table 1, Fig 3C). Thus, all three versions of the analysis yield the same conclusion: that the PCI domains are a group of HEAT domains that have acquired a WH subdomain (the scenario shown schematically in Fig 1B, above).

**Fig 4:**
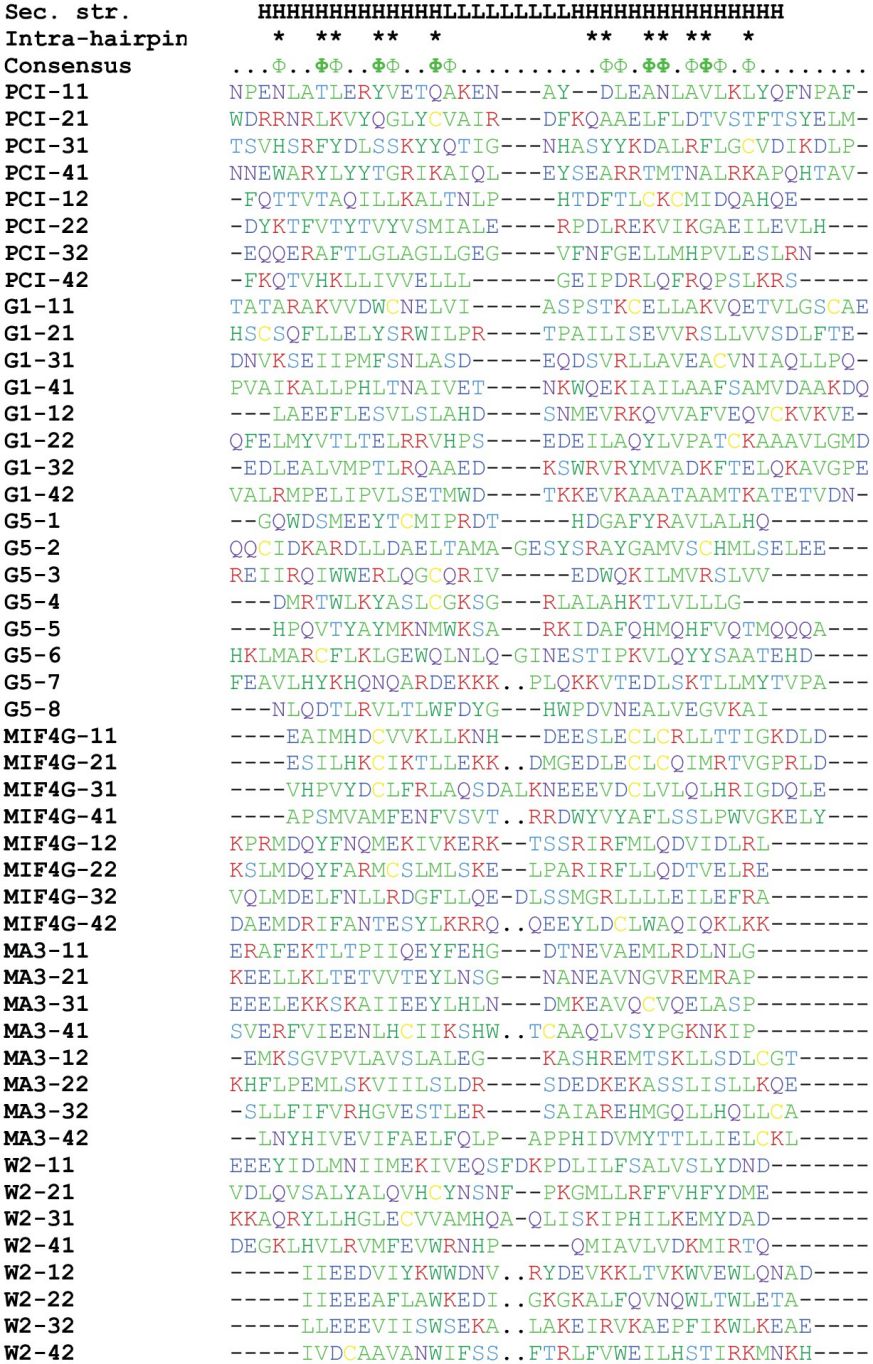
Structure-based sequence alignment of hairpins from HEAT and PCI domains. Eight hairpins per group were used for the alignment. Alignment viewer (https://alignmentviewer.org/) with Clustal residue coloring scheme was used, instead of conservation, because there were no detectable sequence conservation patterns, besides hydrophobic side chains at buried positions. Secondary structure is shown above the alignment: H, helix; L, loop. Positions of intra-hairpin contacts are marked with a “*” above the alignment. Note that side chains at the same position in the alignment may contact both the other helix in the hairpin and an adjacent hairpin, or contact one or the other in different hairpins, due to variations in inter-helix angles and orientations. Positions with hydrophobic side chains in >50% of sequences are labeled with “Φ”; positions with hydrophobic side chains in >75% of the sequences are labeled with “**Φ**” in bold. Gaps are marked with “-“. Positions of deleted amino-acid sequences in the interhelix loop are marked with “‥”.

Structure-based sequence alignment of helical hairpins from PCI and HEAT domain structures showed a preference for hydrophobic side chains at positions buried at the interface between the two helices in the hairpin and, to a lesser extent, at positions facing adjacent hairpins, as expected (Fig 4). No obvious signature motifs could be observed at specific hairpin positions within groups or among the entire alignment, at least with the set of hairpins used here.

## Discussion

The comparative structure similarity analysis of HEAT and PCI domain structures presented here shows that the PCI domains are members of the HEAT domain family. The canonical HEAT domains (Group 1) have similar structure similarity with the MIF4G (Group 3) and PCI (Group 6) domains. And in fact, the PCI domains appear more closely related to the canonical HEAT domains than do the MA3 (Group 2) and W2 (group 4) domains (Fig 3). Sometimes described as atypical HEAT domains [12] and found mostly in eukaryotic translation initiation factors and proteins involved in translation regulation and ribosome biogenesis, the MA3 and W2 HEAT domains tend to be shorter, some with as few as four helical hairpins, often arranged in tandem, and likely evolved from a common ancestor containing a MIF4G, an MA3, and a W2 domains in a row, as observed in eIF4G and CBP80 (reviewed in [34–37]).

To avoid possible score bias due to the length of individual domains, we repeated the structure similarity analysis using four-hairpin fragments of the proteins, which corresponds to the size of the smallest HEAT domains. The results with four-hairpin fragments confirm the results obtained with the intact domains. Using the four-hairpin fragments has its own caveats, because, while for the PCI domains, using the last four hairpins before the WH subdomain ensures that corresponding fragments are used, this is not guaranteed to be the case for the various HEAT domains, even if using the last four hairpins of each protein. However, the observation that the evolutionary trees and groups obtained using intact domains vs. four-hairpin fragments are very similar, supports the validity of this approach. Importantly, both the analysis using intact domains, and that using four-hairpin fragments yield the same results, which strongly supports the overall conclusions.

**Fig 5:**
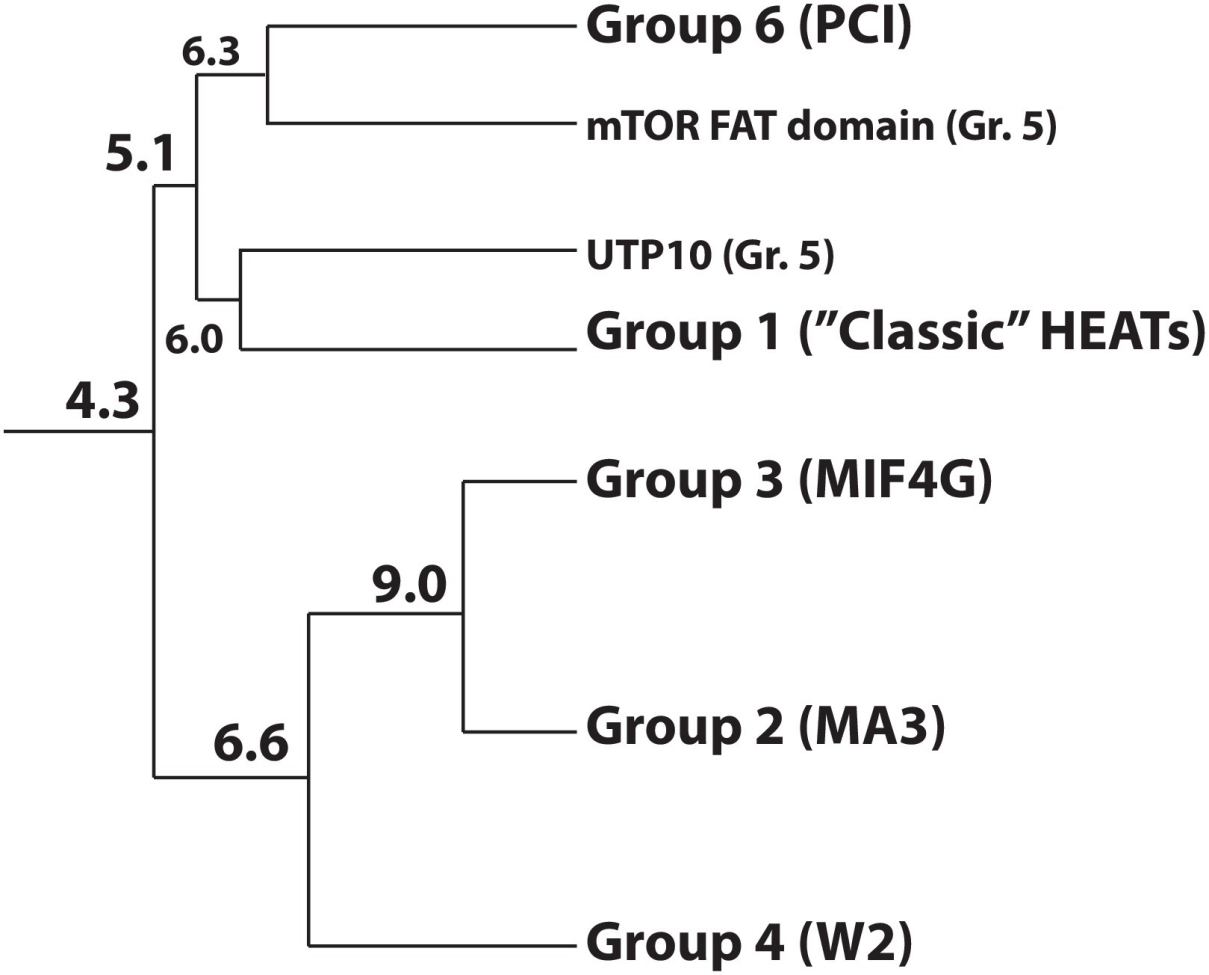
Possible evolutionary tree of HEAT and PCI domains. The dendrogram is based on average structure similarity Z scores from the DALI server [27] between individual groups of domains and branches, using intact HEAT domains and WH-less PCI domains. The outliers, UTP10 and the FAT domain of mTOR, which had been operationally grouped together as Group 5 in Fig 3, Table 1, and Table in S1 Table, had slightly lower structure similarity between each other (Z score 5.7) than their highest average Z scores with other groups (UTP10, Z score 6.0 with Group 1; and the mTOR FAT domain, Z score 5.9 with Group 6). Therefore, these two structures were not considered as a group in building the dendrogram and were added separately, instead, and are shown in smaller font.

The MIF4G and MA3 domain groups are evolutionarily closer to each other than to the rest of the HEAT domains, while the W2 domains are closer to the MA3 domains that to the rest of HEAT domains, including the MIF4G group (Fig 3). These observations indicate that the MA3 and W2 domains could have resulted from the duplication of a MIF4G domain (Fig 5), as opposed to the breaking up of a long HEAT domain solenoid into three consecutive shorter HEAT domains. The propensity of these short HEAT domains to duplicate is illustrated by UPF2, which has three tandem MIF4G domains, and Pdcd4, which has two MA3 domains [38]. eIF5, eIF2Bε, and 5MP/BZW all have W2 domains closely homologous to the W2 domain of eIF4G [29, 37, 39, 40]. 5MP/BZW also has a predicted MA3 domain N-terminal to the W2 domain (refs. [37, 41] and AlphaFold [42, 43]). The much lower structure similarity scores of Group 1 HEAT domains with MA3 and especially W2 domains, compared to MIF4G domains (Table 1, Fig 3), would then suggest different divergence rates of individual groups of domains. As stated in the Introduction, considering the simple, repetitive nature of the helical hairpins, convergent evolution cannot be excluded because there is no detectable sequence similarity between the different groups of domains. The structure of the mTOR FAT domain has average Z score 6.3 with WH-less PCI domains (Fig 5) and 5.9 with intact PCI domains (see **Table in** S1 Table), which is higher than with any of the groups of HEAT domains (the highest average Z score is 4.4 with Group 1, see **Table in** S1 Table). Therefore, it appears that the FAT domains could be more closely related to the PCI domains than to any of the current groups of HEAT domains. However, the absence of detectable sequence similarity makes it impossible to make any reliable conclusions.

In conclusion, this study offers insights into the evolution of the HEAT domain family in eukaryotes and shows that the PCI domains are in fact HEAT domains, which have acquired a WH subdomain at their C-terminus. This further expands the number of proteins containing a HEAT domain, and the already wide range of functions performed by these domains. It would be interesting to trace the origins and divergence of all helical repeat domain protein families as a whole, throughout the evolution of eukaryotes.

## Methods

### Structure similarity searches

The DALI Server [27] was used to search the RCSB PDB database [44] for similar structures and obtain structural similarity scores (Z-scores). The goal was to assemble a diverse set of HEAT and PCI domain structures, as well as identify potential new structures belonging to these families. Searches were initiated with several known HEAT domain and PCI domain structures. An initial diverse set of structures was assembled, eliminating structures with high similarity, as well as selecting high-resolution structures. Each structure and the corresponding structure-based sequence alignment were inspected manually, e.g., to confirm whether a putative PCI domain indeed contains the obligate WH subdomain, or that the DALI-generated automatic structure alignment does indeed define a contiguous helical hairpin structure segment. PyMol [45] was used for structure visualization.

### Structure classification

Any non-HEAT or PCI domain portions of the structures were deleted at this stage using PyMol [45]. Structure similarity scores were calculated for all pairs of structures in the dataset, followed by a second round of removing highly similar structures, yielding the final dataset (**Table in** S1 Table). Where DALI failed to detect similarity, we did not use the pair in future analyses, because the structures are in fact similar. Using the pairs with a Z score 0, or with a Z score 2 (the minimum score considered statistically significant) did not affect the conclusions.

The evolutionary tree automatically generated by the DALI server served as a starting point for grouping the structures in the dataset. The HEAT domains fell into four groups, “classic” HEAT domains, MA3 domains, MIF4G domains, and W2 domains, all of which had previously been defined [5, 12–14, 28–30, 46]. Two HEAT domain structures were not part of any of the groups and were assigned a separate group (Group 5). The PCI domains formed one group (Group 6). The auto-generated dendrogram was correct in most cases, except grouping CTIF3 with Group 3 (MIF4G), instead of Group 1 (“classic” HEAT domains), and grouping the CBP80 MIF4G domain with Group 2 (MA3), instead of Group 3 (MIF4G). In both cases, the Z scores in **Table in** S1 Table unambiguously assign these two structures to the correct group.

### Analysis of structure similarity and evolutionary relationships

We calculated average structure similarity scores (Z scores) and standard deviations for every pair of groups from the pairwise Z scores between all members of the two groups, obtained using the DALI server [27]. The average Z scores between groups of HEAT domains were compared to those between a HEAT domain group and the PCI domains, in order to determine whether or not at least one of the groups of HEAT domains shows greater, or at least comparable, similarity to the PCI domains than to at least one other group of HEAT domains.

To evaluate the contribution of the WH subdomain in the PCI domains, we repeated the analysis using “wingless” PCI domains. The WH subdomains were deleted from the corresponding structures in PyMol [45]. To account for the fact that different HEAT and PCI domains vary greatly in size, we generated four-hairpin fragments from each structure in the dataset and repeated the analysis with those. MOLMOL [47] was used to create figures of structure alignments between HEAT and PCI domains. The dendrogram in Fig 5 was generated using averaged DALI server [27] generated Z scores for intact HEAT domains and WH-less PCI domains. The outliers, UTP10 and the mTOR FAT domain, which had been operationally grouped together (Group 5) to simplify analysis, had relatively modest similarity between each other (Z score 5.7), which was comparable, and even slightly lower than their highest average Z scores: with Group 1 (UTP10, Z score 6.0) and with Group 6 (mTOR FAT, Z score 5.9), respectively. Therefore, these two structures were not considered as a group in building the dendrogram and were added separately, instead.

### Structure-based sequence alignments

Exhaustive PSI-BLAST [19] and HHblits [20] searches yielded statistically significant sequence similarity between proteins belonging to the same group, but not between groups (data not shown). Therefore, we relied on structure alignments in aligning both consecutive hairpins from the same structure and from proteins belonging to different groups. Eight hairpins per group were used for the alignment. The structure alignments were done using the DALI server [27] (larger structures) and in PyMol [45] (hairpins) because the hairpins were too short for the DALI server to recognize statistically significant structure similarity. The structure-based alignment of corresponding hairpins from structures belonging to the same group (where sequence similarity could be observed), was consistent with the PSI-BLAST [19] and HHblits [19] based sequence alignments. We used the Clustal residue coloring scheme, instead of conservation, because there were no detectable sequence conservation patterns, besides hydrophobic side chains at buried positions. Fig 4 was generated with the help of Alignment viewer (https://alignmentviewer.org/).

## Supporting information

S1 Table Pairwise structure homology scores for all structures included in the analysis. (XLSX)
